# Improving frailty assessment: the task is not finished

**DOI:** 10.1186/s13054-023-04477-8

**Published:** 2023-06-01

**Authors:** Bernhard Wernly, Hans Flaatten, Susannah Leaver, Bertrand Guidet, Christian Jung, COVIP investigators, Jesper Fjølner, Michael Beil, Sandra Oeyen, Wojtek Szczeklik, Muhammed Elhadi, Sigal Sviri, Dylan deLange, Rui Moreno, Antonio Artigas, David Dudzinski, Nicolas Serck, Helene Korvenius Nedergaard, Iman Shaat, Aliae Mohamed Hussein, Mostafa Zanaty, Ebtisam Hassanin, Aliae Mohamed Hussein, Nouralsabah Mohamed, Marwa Omar, Ghada Atef Ali Abd El-wahed, Shimaa Touny, Avinash Aujayeb, Saad Nseir, Thomas Urbina, Pierre Garcon, Jean-Philippe Rigaud, Thierry Vanderlinden, Xavier Valette, Buno Megarbane, Elodie Baron, Olivier Nigeon, Gaetan Plantefeve, Camille Foucault, Mehran Monchi, Kristina Fuest, Raphael Bruno, Malte Kelm, Hans-Joachim Kabitz, Stefan Schaller, Abdurraouf Abusalama, Hussein Embarek, Mohamed Anaiba, Ahmed Taher, Akram Alkaseek, Mirjam Evers, Willem Dieperink, Alexander Daniel Cornet, Filipa Brochado, Sonia Lopez-Cuenca, Mohammad Aldiabat, Mohammed Al-Sadawi

**Affiliations:** 1 Department of Internal Medicine, General Hospital Oberndorf, Teaching Hospital of the Paracelsus Medical University Salzburg, Salzburg, Austria; 2 Center for Public Health and Healthcare Research, Paracelsus Medical University of Salzburg, Salzburg, Austria; 3 Department of Research and Development, Haukeland University Hospital, and University of Bergen, Bergen, Norway; 4 General Intensive Care, St. George’s University Hospital NHS Foundation Trust, London, UK; 5 Service de Réanimation Médicale, Hôpital Saint-Antoine, Assistance Publique Hôpitaux de Paris, Paris, France; 6 Medical Faculty, Department of Cardiology, Pulmonology and Vascular Medicine, Heinrich-Heine University Düsseldorf, Moorenstraße 5, 40225 Duesseldorf, Germany

Dear Editor,

We would like to express our sincere gratitude for the thoughtful comments made by Cheung et al. in their response to our letter [1]. We evaluated the distribution and prognostic relevance of previously proposed surrogate parameters for frailty, Clinical Frailty Scale (CFS) [2–4] and the FRAIL checklist [5], in our database and found that in the univariate analysis, both were associated with 90-day mortality. However, after multivariable adjustment for age, gender, SOFA score, and the presence of therapy goal limitations, only the CFS, but not the FRAIL checklist, was still associated with mortality. We concluded that the CFS has added value compared to the FRAIL checklist. Cheung et al. pointed out that the rate of patients with CFS > 4 was higher than those with FRAIL > 0. We agree with Cheung et al.'s assessment that the items of the FRAIL checklist are less concrete than the pictograms of the CFS, and this could be a reason for this discrepancy. Furthermore, Cheung et al. correctly noted that the FRAIL checklist was primarily designed to evaluate patients concerning a modern form of therapy goal limitations, specifically time-limited trials (TLTs). Based on these considerations, Cheung et al. suggested 1) modifying FRAIL and automatically rating the "F" component as "positive" in patients with CFS > 4 in the data analysis which represents post-processing of the data obtained and 2) comparing the rates of TLTs in patients with CFS > 4 and FRAIL > 0. We 1) modified FRAIL according to the proposal, reclassifying 11 patients. These modified-FRAIL > 0 patients again showed excess mortality in the univariate analysis (HR 1.52 95% CI 1.07–2.16; *p* = 0.02). After adjusting for age, gender, SOFA score, and the decision to withdraw/withhold treatment, the modified-FRAIL > 0 was no longer associated with 90-day mortality (aHR 1.15 95% CI 0.80–1.62; *p* = 0.45), confirming previous data and in contrast to a robust multivariable association of CFS > 4 (aHR 1.80 95% CI 1.29–2.53; *p* = 0.001) with mortality. We cannot directly compare the frequency of TLTs since these data were not collected. Of note, the decision to withdraw/withhold treatment in patients with CFS > 4, FRAIL > 0, and modified-FRAIL > 0 was exactly 36% in all three groups.

We summarise that both the CFS and FRAIL are associated with the frequency of withholding treatment, but only the CFS is independently associated with mortality. However, we recognise that the FRAIL checklist attempts to integrate pre-existing conditions and hospitalisations with functional impairment and believe that it is possible that such an approach could further improve frailty assessment.

## Data Availability

The datasets analyzed during the current study are not publicly available due to contractual restrictions but are available from the corresponding author on reasonable request.
